# Core signature of rejection-associated cytokines and chemokines in endomyocardial biopsies after heart transplantation

**DOI:** 10.3389/fcvm.2025.1612258

**Published:** 2025-08-08

**Authors:** Lena M.-L. Radomsky, Jenny F. Kühne, Kerstin Beushausen, Jana Keil, Ludmilla Knigina, Yves Scheibner, Adelheid Görler, Arjang Ruhparwar, Fabio Ius, Christoph L. Bara, Christine S. Falk

**Affiliations:** ^1^ Institute of Transplant Immunology, Hannover Medical School, Hannover, Germany; ^2^ Department of Cardiothoracic, Transplantation and Vascular Surgery, Hannover Medical School, Hannover, Germany; ^3^ German Center for Infectious Diseases (DZIF), TTU Infection of the Immunocompromised Host, Partner Site Hannover-Braunschweig, Hannover, Germany

**Keywords:** heart transplantation, rejection, myocardial biopsies, cytokines, chemokines

## Abstract

**Background:**

Rejection remains a limiting factor for survival after heart transplantation (HTx), and predictive biomarkers are still missing. Therefore, we aimed to define the cytokine/chemokine microenvironment in endomyocardial biopsies (EMB) and plasma after HTx and to identify patterns that reflect ischemia/reperfusion injury as well as allograft rejection. Therefore, we hypothesize distinct cytokine/chemokine patterns in heart biopsies with histopathologically proven rejection compared with the microenvironment in unsuspicious biopsies.

**Methods:**

EMB (*n* = 181; *n* = 52 patients) and peripheral blood samples (*n* = 147; *n* = 52 patients) were obtained between 6 days and 5 years after HTx. 50 immune proteins in EMB tissue lysates and plasma were quantified, and concentrations were compared between EMB with and without histopathologically defined acute rejection (AR), and correlation analyses between tissue and plasma were performed.

**Results:**

Regarding rejection status, distinct cytokine/chemokine patterns were identified with significantly higher concentrations of CCL4, CXCL9, and CXCL10 in EMB with acute rejection (*p* < 0.001). In addition, we identified individual long-term dynamics of patients after HTx associated with rejection. Elevated chemokine concentrations were also detected in EMB of patients with donor-specific antibodies (DSAs). Moreover, significantly different patterns were observed between heart tissue and plasma without direct correlations.

**Conclusion:**

A core signature was defined for EMB with histopathologically proven AR, consisting of high concentrations of CXCL9, CXCL10, CCL3, and CCL4. This EMB chemokine signature was clearly distinct from plasma samples, arguing for a local protein microenvironment associated with AR. Further research is also needed with the help of AI to translate the different approaches for the detection and prediction of AR into clinical practice.

## 1 Introduction

Heart transplantation (HTx) remains the ultimate treatment option for patients suffering from terminal heart failure. Despite enhanced immunosuppressive regimens after HTx, acute rejection (AR) represents a major cause of death during the first years posttransplantation and, hence, affects long-term patient and allograft survival (1–4). Due to rather unspecific symptoms of heart failure, the diagnosis of AR in transplant recipients remains challenging (1). Usually, AR is diagnosed based on the histopathological findings according to the International Society for Heart and Lung Transplantation (ISHLT) classification of an endomyocardial biopsy (EMB) (5, 6). However, the procedure is criticized for lacking objectivity and dependence on the experience of the respective pathologist (7–9). Finally, EMB procurement is an invasive intervention afflicted with medical risks, i.e., perforation, prolonged bleeding, or arrhythmias (10), and may cause tricuspid regurgitation. Therefore, research is also focused on the improvement of, as well as alternatives to, the EMB procedure. Among those, and to circumvent intra- as well as interobserver variability, machine learning (ML) algorithms have been tested for automated interpretation of biopsy-based gene expression profiles (11–13) or whole-slide EMB images (14). However, these promising approaches are not yet part of the clinical routine. Moreover, *in situ* mRNA expression of genes reflecting the allograft rejection in EMBs as well as gene expression profiling and miRNA measurements in blood or plasma, known as liquid biopsies, represent promising tools to improve the diagnosis of AR (8, 9, 15–21).

To the best of our knowledge, and in contrast to other studies employing transcriptional analyses, there is no data available on the signature of soluble immune proteins (SIP), i.e., cytokines, chemokines, and adhesion molecules in lysates of EMBs with or without signs of AR. Here, we could identify a core signature for EBMs with histopathologically proven AR consisting of increased concentrations of the chemokines CXCL9, CXCL10, CCL3, and CCL4. We argue that EMBs are still essential for the diagnosis of AR since there was no correlation between immune protein concentrations in plasma and EMB tissue. Our results could improve understanding of the underlying inflammatory processes in the endomyocardial tissue during AR, to optimize its classification and to identify novel targets for immunotherapy of acute heart allograft rejection.

## 2 Materials and methods

### 2.1 Patients

Overall, 52 patients were included with a total of 181 EMBs at different time points post HTx in our biopsy cohort and 52 patients counting 147 samples in the plasma cohort, with a few patients (*n* = 17) appearing in both. The mean patient age was 49.4 years with 69% male recipients in the HTx biopsy cohort and 50.1 years with 73% male recipients in the plasma cohort (Table 1). All patients provided written informed consent approved by the ethics committee of Medizinische Hochschule Hannover (MHH) (7913_BO_S_2018). Cardiac diagnoses for HTx were: dilated cardiomyopathy (DCM; 34.6%), infected left ventricular assist device (LVAD; 30.8%), ischemic cardiomyopathy (ICM; 9.6%), and retransplantation (ReHTx; 3.9%). Patients received an immunosuppressive therapy with steroids, calcineurin inhibitors (tacrolimus, cyclosporine A), mycophenolate mofetil (MMF), or an mTOR inhibitor (everolimus). Regarding DSAs, 4 biopsies (out of 181, 2.2%) were pathologically graded as AMR1, and all remaining 177 biopsies were AMR0. Nearly one-fifth of the HTx recipients (19.2%) were donor-specific antibodies (DSA)-positive after HTx. Some patients were treated with antithymocyte globulin (ATG, *n* = 27, 14.9%) and/or IL-6 receptor antibody, tocilizumab (*n* = 7, 3.9%). We could not detect significant clinical differences between the plasma and biopsy cohorts shown in Table 1. Donor hearts were retrieved from DBD (donation after brain death) organ donors. After explantation and cold flush with histidine–tryptophan–ketoglutarate solution (HTK; Custodiol®, Essential Pharmaceuticals LLC, Durham, NC, USA), hearts were either stored in cold HTK [standard of care (SOC)] or perfused by normothermic oxygenated *ex situ* heart perfusion (ESHP) (Organ Care System™; TransMedics®, Andover, MA) (Table 1).

**Table 1:** Patient demographics and clinical characteristics.

Characteristics	Biopsy cohort	Plasma cohort
Patients	*N* = 52	*N* = 52
Samples	*n* = 181	*n* = 147
Age at Tx (years)	49.38 ± 13.18	50.12 ± 13.99
Gender (sex), patients (%)		
Male	36 (69.23%)	38 (73.08%)
Female	16 (30.77%)	14 (26.92%)
Transplant indication, patients (%)		
DCM	18 (34.62%)	20 (38.46%)
ICM	5 (9.62%)	9 (17.31%)
LVAD infect	16 (30.77%)	14 (26.92%)
ReTx	2 (3.85%)	2 (3.85%)
Others	11 (21.15%)	7 (13.46%)
Immunosuppressive medication, patients (%)		
Steroids	52 (100%)	47 (90.38%)
Tacrolimus	48 (92.31%)	45 (86.54%)
Cyclosporine	5 (9.62%)	4 (7.69%)
Mycophenolate mofetil	46 (88.46%)	42 (80.77%)
Everolimus	26 (50%)	22 (42.31%)
Antithymocyte globulin	33 (63.46%)	36 (69.23%)
Tocilizumab	7 (13.46%)	8 (15.38%)
Donor-specific antibodies (DSA), patients (%)		
Yes	10 (19.23%)	
No	23 (44.23%)	
Unknown	19 (36.54%)	
Bridge to transplant, patients (%)	40 (76.92%)	39 (75%)
Organ preservation, patients (%)		
SOC	28 (53.85%)	26 (50%)
ESHP	24 (46.15%)	22 (42.31%)
CIT (min)	167.5 ± 65.65	176.6 ± 72.68
Rejection grade, biopsies (%)	(*n* = 181)	(*n* = 47)
0R	121 (66.85%)	37 (78.72%)
1R	58 (32.04%)	10 (21.28%)
2R	2 (1.1%)	0 (0.0%)
Donor characteristics		
Donor age (years)	43.94 ± 12.76	41.48 ± 48
Donor gender male	25 (48.08%)	30 (57.69%)
Donor gender female	27 (51.92%)	22 (42.31%)

No significant (*p* < 0.05) differences could be detected between plasma and biopsy cohorts (unpaired *t*-test, Mann–Whitney test). Unpaired *t*-test (Mann–Whitney test) was applied to compare the demographic and clinical data of the plasma and biopsy cohorts. Age and CIT data are mean values ± standard deviation (SD). Tx, transplantation; ReTx, retransplantation; DCM, dilative cardiomyopathy; ICM, ischemic cardiomyopathy; LVAD, left ventricular assist device; SOC, standard of care; ESHP, *ex situ* heart perfusion; CIT, cold ischemic time.

### 2.2 Endomyocardial biopsies (EMBs)

EMBs (*n* = 181 of *n* = 52 patients) were taken either per protocol or indication (6 days up to 5 years after HTx) from the right ventricle of the heart, representative of the ventricular myocardium. At the time of EMB procurement, no signs of active inflammation/infection were clinically detectable. Rejection status was histopathologically determined according to ISHLT classifications ranging from 0R to 3R (5, 6). EMBs were stored at −20°C until further processing. Protein lysates were prepared using Bio-Plex Cell Lysis Kit (Bio-Rad Laboratories, Hercules, CA, USA). Briefly, the tissue samples, each consisting of one EMB, were mixed with lysing solution and stored at −80°C overnight. The next day, samples were thawed and sonicated for 10 min. After centrifugation for 20 min at 4°C and 13,000 rpm, the supernatant was collected without disturbing the pellet. The resulting lysates were stored at −20°C.

Total protein concentrations of the lysates were measured using the Pierce™ BCA Protein Assay Kit (Thermo Fisher Scientific, Rockford, IL, USA), according to the manufacturer's instructions. EMB protein lysates were normalized to a total protein concentration of 250 or 500 µg/ml, respectively, for subsequent quantification of soluble immune proteins (see Section 2.4).

### 2.3 Peripheral blood samples

Peripheral blood samples of *n* = 52 HTx recipients were obtained at different time points after HTx (starting prior to HTx up to 5 years post HTx). If possible, the time point of plasma sample obtainment was matched with biopsy procurement (±8 days). EDTA plasma was frozen after centrifugation (1,500 rpm for 15 min at room temperature) at −20°C until cytokine/chemokine quantification. Total protein concentrations were not measured for plasma samples.

### 2.4 Cytokine and chemokine quantification

Concentrations of 50 soluble immune proteins (SIP) were determined by Luminex-based multiplex technology (Bio-Plex Pro Human Cytokine Panel #010420, Bio-Rad Laboratories, Hercules, CA, USA), according to the manufacturer's instructions (22, 23). EMB protein lysates were normalized with sample diluent to a total protein concentration of either 250 or 500 µg/ml, with 50 µl (=12.5/25 µg total protein) used for the assay. The respective total protein concentrations were taken into account for the calculation of the absolute cytokine/chemokine concentrations. Plasma samples were diluted 1:2 with sample diluent. Bio-Plex Manager 6.1 software (Bio-Rad Laboratories) was used to calculate standard curves and concentrations.

### 2.5 Statistical analyses

Statistical analyses were performed by GraphPad Prism Software (Versions 7 and 9, La Jolla, CA, USA). D’Agostino–Pearson omnibus normality test was used to assess data distribution. According to data distribution, comparisons of two groups were calculated using suitable unpaired *t*-tests (either Kruskal–Wallis or Mann–Whitney test). For paired analyses, the Wilcoxon test was applied. Moreover, the receiver operating characteristic curve and area under the curve were calculated for the prediction of rejection status based on cytokine/chemokine concentrations.

Furthermore, the dataset was analyzed using Qlucore Omics Explorer software (Version 3.5, Lund, Sweden). Therefore, data were log2 transformed and scaled to mean = 1, variable = 0, and threshold of 0.01. For cluster analyses, principal component analysis (PCA) and unsupervised hierarchical clustering (UHC) analysis were generated. To identify the immune mediators that differed most significantly between the experimental groups, two-group or multigroup comparisons were performed. Additionally, volcano plot analysis and KNN analyses were applied. Statistical tests, *q*- and *p*-values are indicated in the figure legends. Significance was considered for *p*-values <0.05.

## 3 Results

### 3.1 Elevated cytokine and chemokine concentrations in EMB lysates during histopathologically proven acute rejection

**Figure 1:**
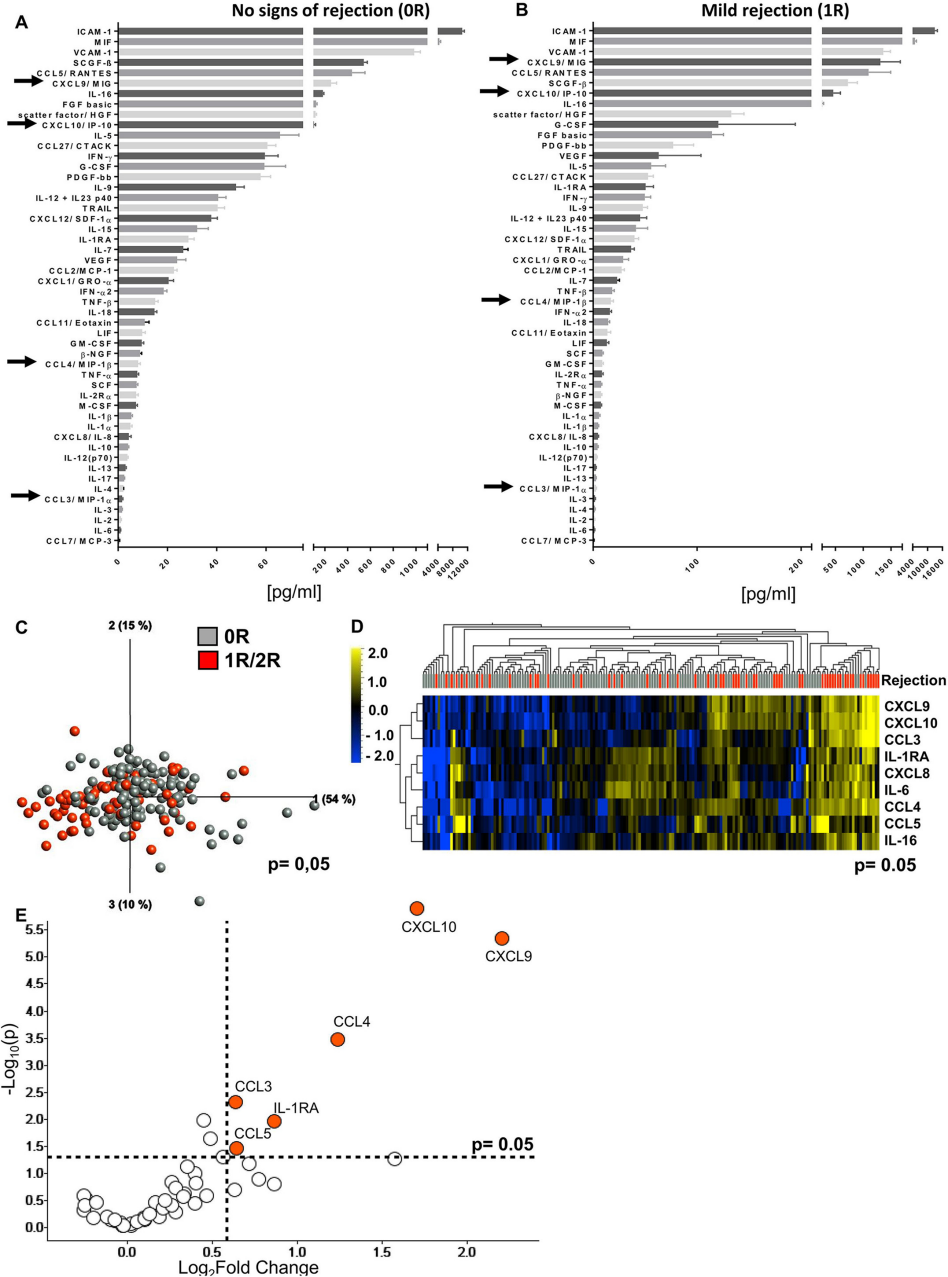
Cytokine/chemokine microenvironment in heart biopsies with no signs of rejection and with acute mild rejection. Biopsies were taken at different time points after heart transplantation (6 days to 5.5 years post Tx) and histopathologically classified as 0R **(A)** or 1R **(B)** according to ISHLT standardized nomenclature (0R, *n* = 121; 1R, *n* = 58). Lysate concentrations of the heart tissue were normalized by adjusting the total protein concentration to 250 or 500 µg/ml and then assessed for concentrations of 50 different soluble mediators by Luminex-based multiplex assays. Waterfall plots depicting protein concentrations from highest to lowest measured concentrations. Important mediators are marked with arrows. **(C)** Principal component analysis of the 50 measured proteins according to 0R or 1R/2R (0R, *n* = 121, gray; 1R/2R, *n* = 60, red) (*p* = 0.05 and *q* = 0.198) and **(D)** unsupervised hierarchical clustering are shown. Two-group comparisons were used to identify variables differentially expressed between the two groups. Blue color indicates lower, and yellow color indicates higher expression. **(E)** The volcano plot shows the difference between 0R and 1R/2R based on fold change (*x*-axis) and *p*-value (*y*-axis). Proteins with log2 fold change ≥0.5 and *p*-value <0.05 are colored and labeled.

To define the microenvironment in heart tissue after HTx, we quantified 50 SIP in EMB lysates (*n* = 181), taken from *N* = 52 patients. In terms of acute rejection (AR), EMBs were classified as no signs of rejection (0R, *n* = 121, 66.9%), mild rejection (1R, *n* = 58, 32.0%), or moderate rejection (2R, *n* = 2, 1.1%) (Table 1) at different time points after HTx. The hierarchy of cytokine/chemokine concentrations was comparable in tissue lysates of 0R vs. 1R biopsies, with few chemokines placed at a different rank and a trend toward higher concentrations in the AR group (Figures 1A,B). These differences became obvious by PCA and UHC analyses, where 1R/2R rejection samples clearly separated from 0R samples, exhibiting significantly lower cytokine/chemokine concentrations (Figure 1C). Nine cytokine/chemokine concentrations were significantly different between 0R and 1R groups (CXCL8–CXCL10, CCL3–CCL5, IL-6, IL-16, and IL-1RA, Figure 1D). Maximum fold change between the groups was detected for CXCL9 and CXCL10 followed by CCL4, IL-1RA, CCL3, and CCL5 (Figure 1E). In addition, we detected high amounts of adhesion molecules, typically expressed by endothelial as well as immune cells such as ICAM-1, VCAM-1, and FGF-basic and molecules involved in tissue repair such as HGF and PDGF-bb (Figures 1A,B). In contrast, rather low concentrations were detected in both groups for inflammatory proteins such as IL-6, CXCL8, or IL-1α and IL-1β. To control for a potential effect of the 2R biopsies on cytokine/chemokine concentrations, we compared 0R samples with only 1R biopsies. This yielded comparable results, indicating no further increase in cytokine/chemokine concentrations due to moderate rejection (Supplementary Figure S1).

**Figure 2:**
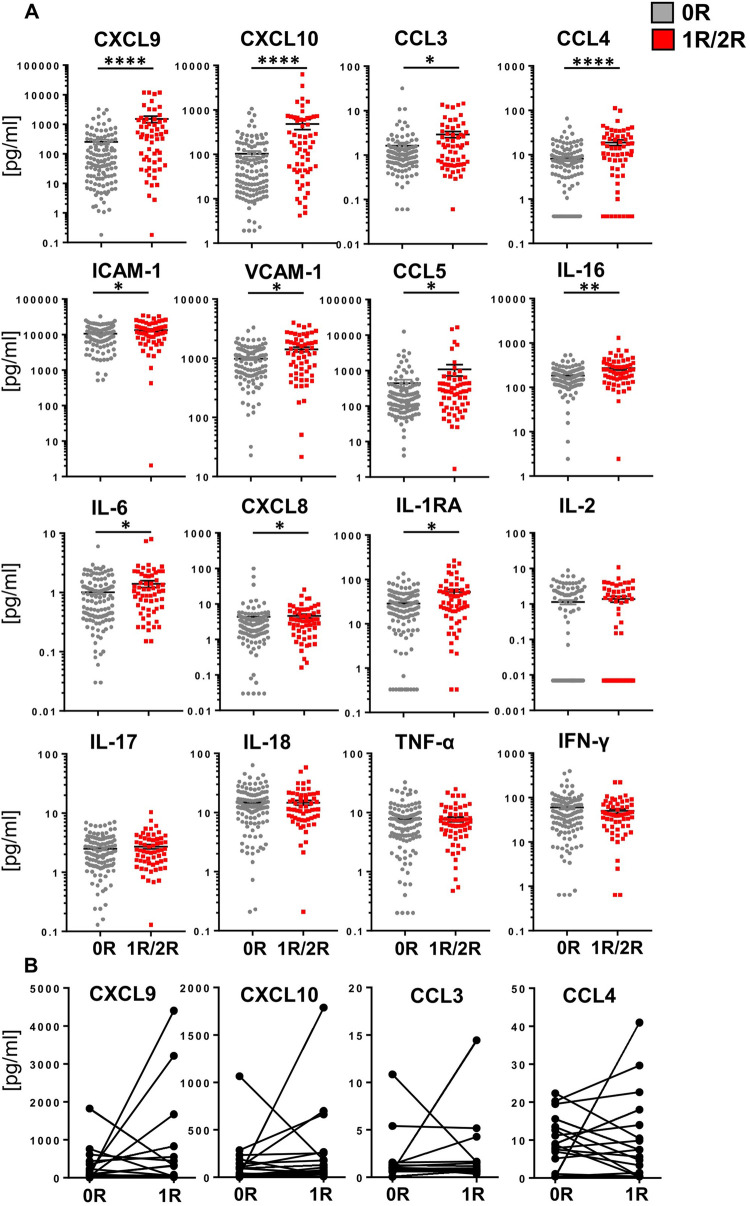
Biopsies obtained during acute rejection reach significantly higher concentrations of some core cytokines and chemokines. Biopsies were taken and cytokine/chemokine concentrations determined as described in Figure 1. **(A)** For statistical analysis, a two-tailed, unpaired *t*-test (Kruskal–Wallis) was applied. Data are shown as mean ± SEM (0R, *n* = 121, gray; 1R/2R, *n* = 60, red). **(B)** Paired *t*-test (Wilcoxon test) was applied on two consecutive biopsies with the second biopsy classified as histopathologic acute mild rejection (1R) (*n* = 17). *p < 0.05 **p < 0.01, ***p < 0.001, ****p < 0.0001.

Comparing the individual protein concentrations of EMBs with histopathologically proven AR and unsuspicious EMBs revealed significantly elevated concentrations for 11 SIP during AR. Particularly, concentrations of the chemokines CXCL9, CXCL10, CCL3, and CCL4 reached high, significantly increased concentrations in EBMs with AR (*p* < 0.0001) (Figure 2A). Interestingly, the histopathological heterogeneity associated with 1R was reflected as a broad concentration range of most cytokines and chemokines associated, leading to a non-parametric distribution and the application of statistics for non-parametric data sets (Supplementary Table S1). In addition, other cytokines such as IL-6 and IL-16 and chemokines such as CXCL8 and CCL5 as well as adhesion molecules such as ICAM-1 and VCAM-1 were also enriched in EMBs with AR, while similarly low levels of the typical T-cell-derived cytokines such as IFN-γ, TNF-α, and IL-17 were observed. Interestingly, paired analyses of EMBs (*n* = 17) without signs of AR (0R) with a subsequent biopsy classified as 1R did not provide significantly increased cytokine/chemokine concentrations (Figure 2B, Supplementary Figure S2A), although at individual level, several patients displayed elevated chemokine concentrations shortly before or during AR (Supplementary Figures S2B–D).

### 3.2 Definition of a chemokine core signature can differentiate EMBs with and without acute rejection

None of the 50 SIP alone was able to discriminate between 0R and 1R/2R EMB samples (Supplementary Figure S3A). However, KNN analysis identified the combination of the chemokines CXCL9, CXCL10, CCL3, and CCL4 as core signature representing the minimal markers necessary to distinguish between EMBs with or without histopathological signs of AR. Of note, the sum of the four core chemokine concentrations yielded highly significant enhanced chemokine levels in the 1R rejection group, further supporting this pattern (Supplementary Figure S3B).

**Figure 3:**
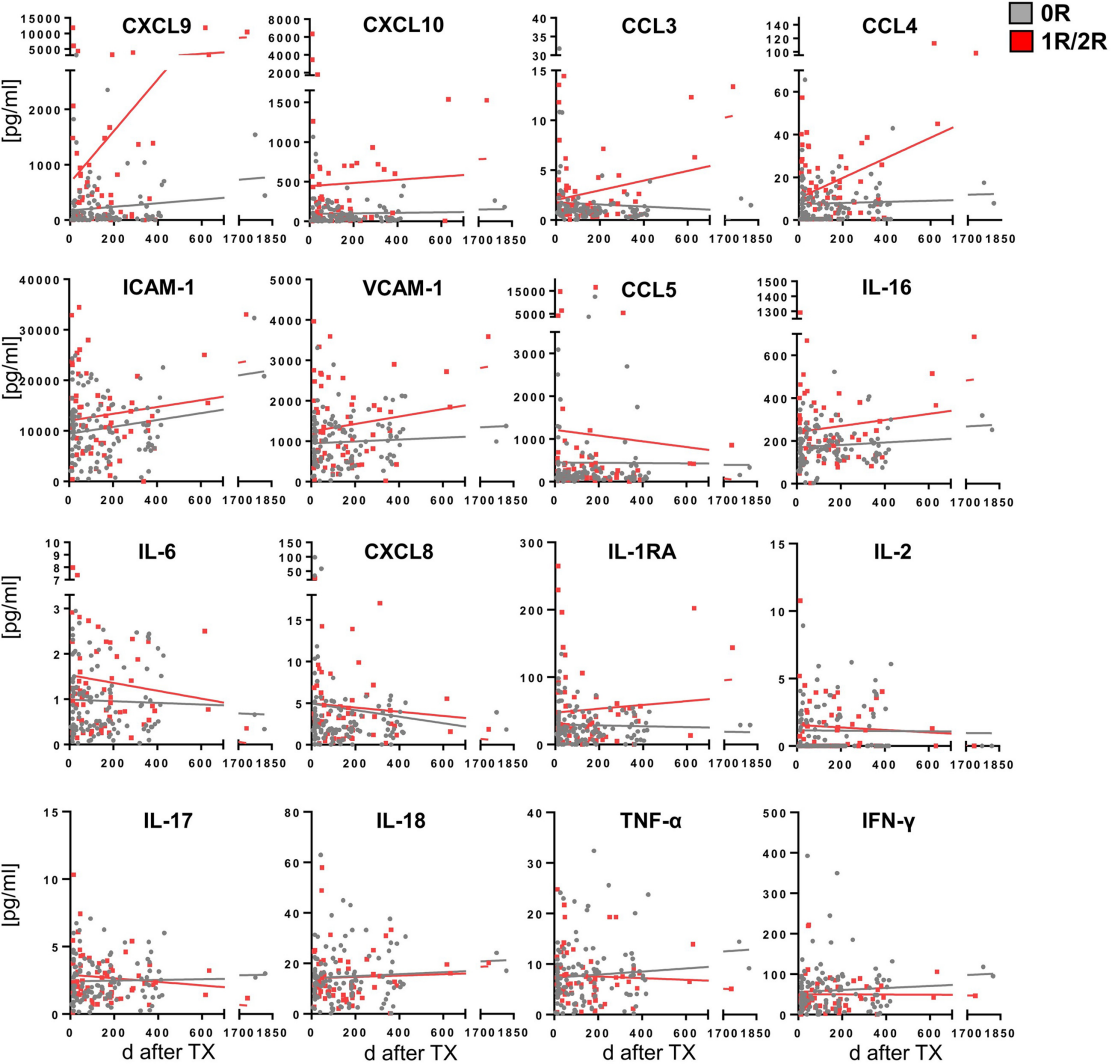
Steady lower cytokine/chemokine concentrations in the no-rejection group over time and higher upwards trending concentrations in the acute rejection group. Biopsies were processed and cytokine/chemokine concentrations quantified as described in Figure 1. Cytokine/chemokine concentrations were correlated to time after Tx (days) for 0R (gray; *n* = 119) and 1R/2R samples (red; *n* = 58), respectively. Linear regression was performed with significant slope derivation from zero for CXCL9, CCL3, and CCL4 concentrations in 1R/2R samples. R2 did not reach values >0.8.

Correlating SIP concentrations with time post HTx revealed a trend toward rising concentrations of most immune proteins for all time points for 1R/2R EMBs compared with the 0R group (Figure 3). Moreover, concentrations of 0R EMBs remained at stable low levels over time, whereas lysates of AR (1R/2R) EMBs showed higher concentrations at later time points. A significant slope derivation from zero in the group of EMBs with AR was found for CXCL9, CCL3, and CCL4, indicating that higher concentrations of these core signature chemokines in EMBs with AR are even more pronounced with time after HTx (Figure 3).

### 3.3 Minor influence of recipient DSA status, tocilizumab or ATG treatment, bridge to transplant, and organ preservation technique on cytokine/chemokine concentrations

**Figure 4:**
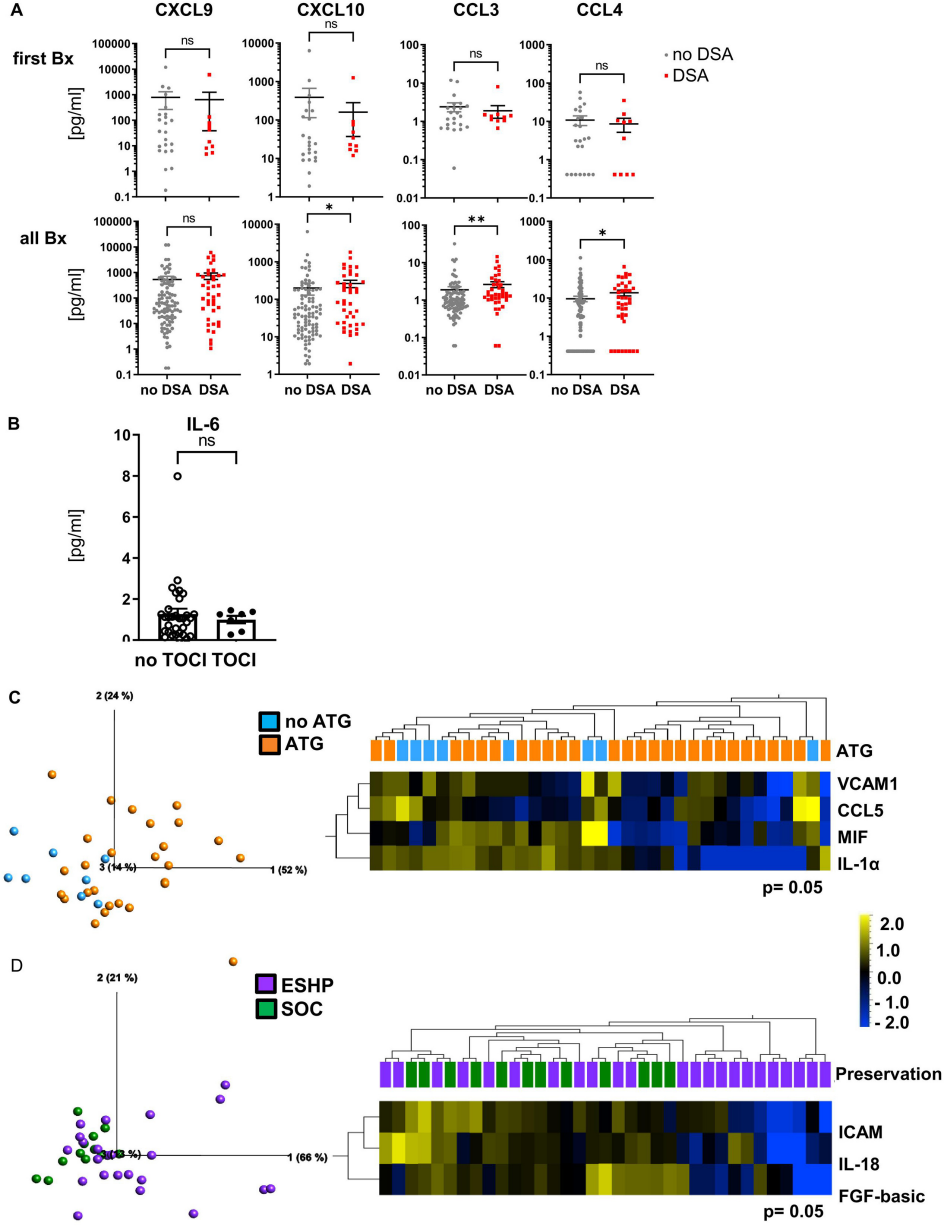
Minor influence of recipient DSA status, tocilizumab or ATG treatment, bridge to transplant, and organ preservation method on cytokine/chemokine concentrations. EMBs were processed and cytokine/chemokine concentrations determined as described in Figure 1. Groups were formed based on clinical patient data such as **(A)** recipient DSA status [no DSA, *n* = 23 (95), gray; DSA, *n* = 10 (41), red for first EMB (all EMB) taken], **(B)** tocilizumab (tocilizumab, *n* = 7, open symbols; no tocilizumab, *n* = 32, filled symbols), or **(C)** ATG treatment (ATG, *n* = 27, orange; no ATG, *n* = 8, blue) and **(D)** preservation method prior to implantation (ESHP, *n* = 23, purple; SOC, *n* = 12, green). **(B–D)** Only the first biopsy per patient obtained during the first 26 days after transplantation was included. For statistical analysis (for **A**,**B**), a two-tailed, unpaired, non-parametric Mann–Whitney test was applied. Data are shown as mean ± SEM. *p < 0.05 **p < 0.01, ***p < 0.001, ****p < 0.0001. **(C,D)** Principal component analysis of the 50 measured proteins (*p* = 0.05 and qB = 0.408; qC = 0.261) and unsupervised hierarchical clustering are shown. Two-group comparisons were used to identify variables differentially expressed between the two groups. Blue color indicates lower, and yellow color indicates higher expression.

Since the presence of circulating donor-specific antihuman leukocyte antigen (HLA) antibodies (DSAs) is regarded to be a required condition for AMR after HTx (24), we first correlated the concentrations of our core chemokines, CXCL9, CXCL10, CCL3, and CCL4, to the recipient DSA status (Figure 4A). When only the first EMB was considered, slight trends toward reduced concentrations of these core signature chemokines were observed in patients with DSA. However, when considering all biopsies, chemokine concentrations were higher in patients with DSA, reaching statistical significance for CXCL10, CCL3, and CCL4.

Next, we analyzed the potential impact of immunosuppressive treatment, bridge to transplant (BTT; i.e., LVAD), and organ preservation technique (SOC/EVHP) on cytokine/chemokine concentrations in EMB lysates during the first month post HTx. No impact of the IL-6 receptor blocking antibody tocilizumab at the time of HTx was detected on IL-6 concentrations (Figure 4B), despite the very low IL-6 concentrations, and also no effect was seen for bridge to transplant (BTT, i.e., LVAD, Supplementary Table S2). Only a minor influence of ATG on cytokine and adhesion molecule concentrations was observed (Figure 4C) with lower concentrations of VCAM-1, MIF, and CCL5 in the ATG group. Furthermore, EMBs of standard of care cold storage (SOC) preserved hearts trended toward higher concentrations of, i.e., ICAM-1, IL-18, and FGF-basic, when considering the first biopsy taken post HTx (Figure 4D). This trend was more pronounced when analyzing all EMBs taken during the first year after HTx (Supplementary Figure S4). Thus, IL-6 receptor blockade with tocilizumab, ATG, BTT, and preservation technique seems to have minor effects on the cytokine/chemokine microenvironment in EMBs and, importantly, did not have an impact on the core signature associated with AR. Our data further suggest that the presence of DSA in recipient blood does not seem to have a strong impact on chemokine concentrations in myocardial tissue in the early phase post HTx but may contribute to the micromilieu at later stages.

### 3.4 Distinct microenvironment in paired plasma samples and biopsy lysates

We previously identified specific inflammatory signatures for ischemic vs. reperfusion phases of HTx in the plasma of heart recipients (18). Extending our analyses by comparing plasma samples of 50 patients before and directly after HTx, we found significantly higher concentrations of various cytokines/chemokines such as IL-6, IL-10, IL-1RA, and IL-18 post HTx, leading to clearly separated clusters in PCA and UHC analyses (Supplementary Figures S5A,B), thereby confirming our previous findings with a larger cohort of plasma samples (17). KNN analysis revealed IL-1RA and IL-10 as major discriminators, sufficient to distinguish between pre- and post-HTx samples and, hence, reflecting ischemia reperfusion injury (IRI).

**Figure 5:**
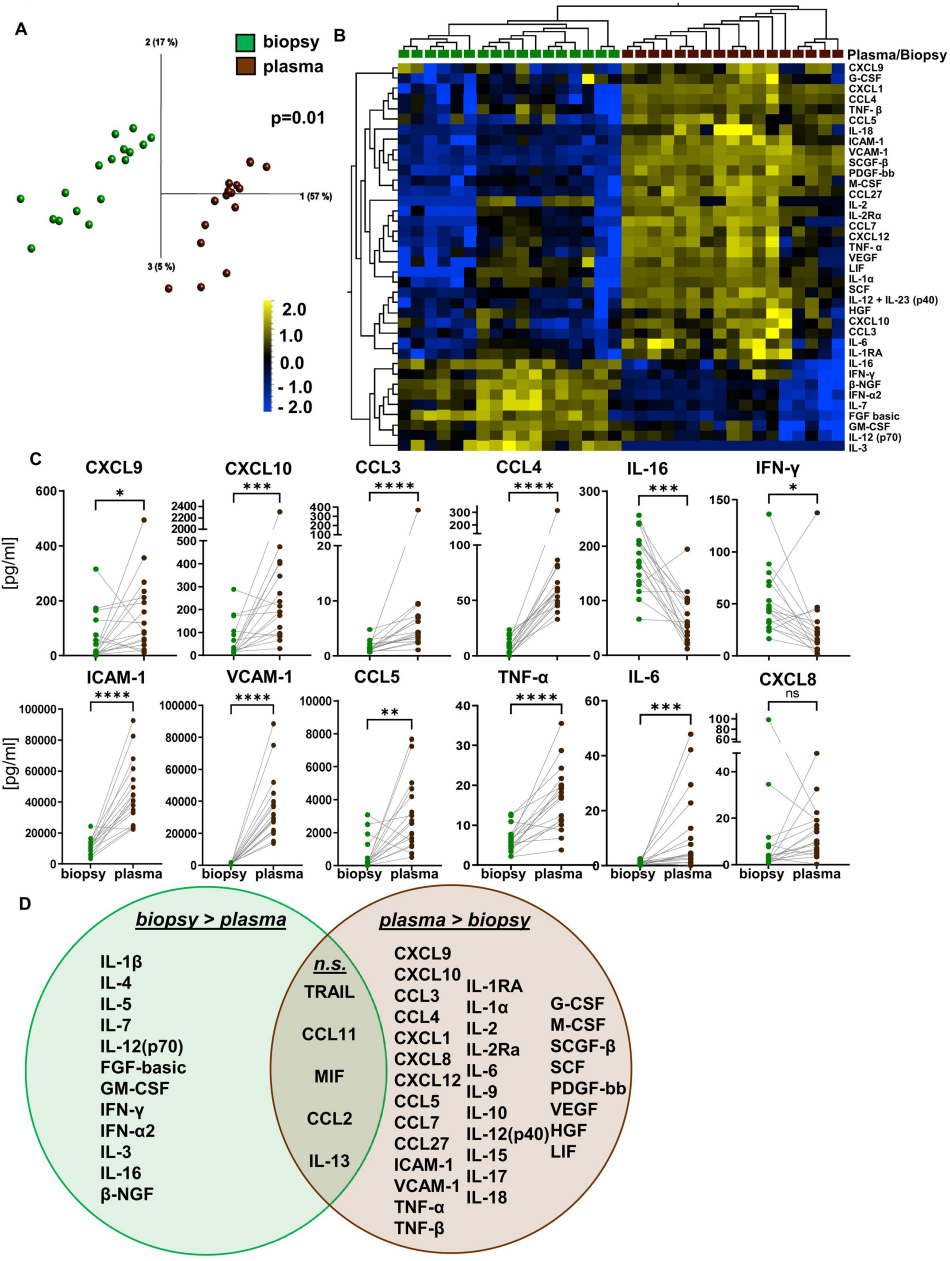
Distinct microenvironment in paired plasma samples and biopsy lysates. Biopsies were taken and cytokine/chemokine concentrations determined as described in Figure 1. Here, only biopsies without histopathologic signs of acute rejection (0R) were included in the analysis and compared with the concentrations of the same 50 proteins quantified in paired peripheral blood plasma samples obtained ± 8 days to EMB procurement (biopsy, *n* = 17, green; plasma, *n* = 17, brown). **(A)** Principal component analysis and **(B)** unsupervised hierarchical clustering of the 50 measured proteins according to sample origin of either plasma or tissue (*p* = 0.01 and *q* = 0.05) are shown. Two-group comparisons were used to identify variables differentially expressed between the groups. **(C)** Two-tailed, paired Wilcoxon matched-pairs rank test was applied. Data are shown as mean ± SEM. *p < 0.05 **p < 0.01, ***p < 0.001, ****p < 0.0001. **(D)** Venn diagram mapping proteins significantly higher concentrated either in plasma or heart tissue lysates. Overlap displays proteins with no significant difference.

To determine whether the micromilieu in EMBs is also detectable in the circulation, we tested whether the relative protein concentrations in EMBs were reflected by similar patterns in paired plasma samples (17). EMBs without signs of rejection (0R) were included with an interval to plasma samples of a maximum of 8 days. Both PCA and UHC revealed a clear separation between the two groups (Figures 5A,B). Even in the absence of rejection (0R), for some of the analytes (i.e., our core signature chemokines), we detected even lower cytokine/chemokine concentrations in EMB lysates as compared with paired plasma. The Wilcoxon matched-pairs signed rank test (two-tailed) revealed statistically significant differences for most of the analytes, such as soluble ICAM-1, VCAM-1, the chemokine CCL5, and the cytokine TNF-α, which is indicative of the distinct microenvironments in these two compartments. In contrast, several cytokines, i.e., the pro-inflammatory molecules IL-16 and IFN-γ, were more concentrated in EMBs compared with paired plasma (Figures 5B–D). These effects were also seen when considering 1R EMB lysates and paired plasma samples, despite the very low sample size (*n* = 6; Supplementary Figure S6). A weak correlation between plasma and tissue lysate concentrations was only found for CCL2, but for none of the other proteins including the core signature chemokines (data not shown). Finally, none of the 50 SIP displayed changes in plasma concentrations during AR, indicating a unique microenvironment in the EMB compartment (Supplementary Table S3). Taken together, the chemokines CXCL9, CXCL10, CCL3, and CCL4 represented a core signature for AR in EMB lysates after HTx, but neither the core signature chemokines nor other soluble factors could hallmark AR in recipient plasma.

## 4 Discussion

Acute rejection remains a major risk for death and worse long-term outcomes after HTx (1–4). Hence, early diagnosis, accomplished by histopathological examination of EMBs, is crucial. Despite certain limitations and attempts to improve the diagnosis of AR, i.e., by detection of miRNA in myocardial tissue or gene expression profiling, EMBs still represent the gold standard for the diagnosis of AR (1, 5, 6, 15, 25–28). However, biomarkers for AR are still missing, and to the best of our knowledge, we are the first to describe the microenvironment in EMB protein lysates based on concentrations of 50 SIP. Interestingly, molecules typically expressed by endothelial cells, such as ICAM-1 and VCAM-1, but also factors involved in cell proliferation or wound healing, such as HGF and PDGF-bb, dominated the myocardial micromilieu, whereas inflammatory proteins such as IL-6 and CXCL8 or IL-1α and IL-1β showed rather low concentrations. Furthermore, we detected significantly higher concentrations during acute mild rejection (1R) as compared with healthy tissue for the chemokines CCL3, CCL4, CXCL9, and CXCL10 that were identified as core signature capable of discriminating between EMBs diagnosed with acute mild rejection (1R) vs. unsuspicious EMBs.

The chemokines CXCL9 and CXCL10 can be induced in all immune, endo- and epithelial cells by IFN-γ, subsequently promoting Th1 immune responses. The CXCR3 ligands CXCL9 and CXCL10 are involved in the migration of alloantigen-primed T cells and other leukocytes into transplanted organs (29, 30). In addition, CXCL10 is expressed by endothelial cells and tissue-infiltrating leukocytes (31). Beneficial effects could be demonstrated for CXCL10-deficient cardiac allografts in mice where they exhibited prolonged organ survival and reduced cytokine/chemokine expression as well as CXCR3 receptor expression in comparison with CXCL10 wild-type allografts (29, 31). CXCR3 receptor expression can be induced on activated T and NK cells as well as malignant B cells (32). In this regard, CXCR3-deficient mice were shown to be resistant against the development of acute cardiac rejection, and treatment with anti-CXCR3 mAbs or non-peptide small molecule inhibitors against CXCR3 induced prolonged murine cardiac allograft survival (29, 33, 34). Even though concentrations of CXCL9 and CXCL10 were significantly elevated in EMBs with AR, we did not observe a simultaneous increase in IFN-γ levels in the rejection group. Eventually in our setting, early inflammation and organ damage may have occurred before EMB procurement; hence, IFN-γ levels were comparable between 0R and 1R/2R groups.

In addition, the core signature for EMBs with AR comprises the chemokines CCL3 and CCL4. While CCR5 is the receptor for both CCL3 and CCL4, CCL3 can also bind to CCR1 which was identified as a key player in the development of cardiac rejection in mice by prolonged allograft survival of CCR1-deficient mice in comparison with wild-type mice (35). CCR5-deficient mice showed prolonged survival as well as persistent acceptance of MHC-II-mismatched cardiac allografts, and treatment with neutralizing monoclonal antibody against CCR5 resulted in improved survival (36). Collectively, these studies and our results underline the importance of CCL3 and CCL4 in the development of rejection (29, 35, 36).

Unfortunately, we could not show higher concentrations of our core signature chemokines during AR for all 1R/2R patients, which may result from distinct immune cell repertoires in heart tissue as well as a certain degree of variability in histopathology (9, 37, 38). Eventually, inflammatory responses were already ongoing prior to EMB procurement and subsequent histopathological diagnosis of AR. However, in a number of patients, individual chemokine kinetics clearly showed elevated chemokine concentrations during or shortly before the onset of AR, suggesting that the individual development of core signature chemokines may be able to support AR diagnostics. The 0R group without signs of rejection displayed stably low concentrations indicating that longer intervals after HTx may not induce physiological changes in the cytokine/chemokine microenvironment in EMBs. A similar observation was published for CXCL9 and CXCL10 in serum samples (39). In contrast, EMB of the 1R group showed constantly higher concentrations of several cytokines/chemokines than the 0R group associated with rising concentrations at later time points of AR diagnosis. This kinetic may indicate an intensified tissue response even during mild rejection at later time points, eventually due to previous local immune responses and/or damage to allograft tissue. In line with our observations, elevated expression of CXCR9, CXCR10, and their receptors was demonstrated at the mRNA level in EMBs during rejection at later time points compared with rejection shortly after HTx (40).

The comparison of the rejection-associated core signature detected in EMBs with AR to the IRI pattern in plasma directly after HTx uncovered an overlap of several cytokines/chemokines, i.e., CCL4, CXCL9, CXCL10, IL-6 or IL-1RA, or CXCL8, with increased concentrations in both compartments. The differences between ischemia and reperfusion responses were already described by our group with increased concentrations of pro- and anti-inflammatory cytokines, chemokines, and soluble adhesion molecules in recipient plasma as characteristic of the reperfusion injury (22). Thus, CXCL9, CXCL10, and CCL4 appear to be increased in plasma after HTx, early as part of IRI and later during acute rejection, most likely mediated by similar tissue injury responses. Of note, KNN analyses identified the anti-inflammatory cytokines IL-10 and IL-1RA as main discriminators for IRI in plasma of HTx recipients (22).

To reduce the frequency of EMBs per patient, so-called liquid biopsies for detection of donor-derived cell-free DNA (dd-cfDNA) have been investigated as a less invasive follow-up procedure after HTx. Dd-cfDNA has been shown to be released in significant amounts during rejection (41–43) supported by gene expression profiling (27, 42).

Here, we compared the microenvironment in EMB tissue with the milieu in paired plasma samples at the time of biopsy. These compartments could be clearly separated by their different cytokine/chemokine patterns, arguing for a local microenvironment within the myocardium. A weak correlation was seen only for CCL2 between plasma and EMB tissue, but not for the core signature chemokines. One study identified plasma levels of CDL5, an apoptosis inhibitor, capable of predicting acute cellular rejection in the first year after HTx (44). Yet, in our plasma analyses, no significant changes were detected during mild AR, also indicating that immune responses leading to AR develop locally in myocardial tissue and may not be represented systemically in plasma. Therefore, EMBs remain essential for the diagnosis of cardiac AR since current diagnostics still seem to be insufficient to completely replace this tissue examination.

Importantly, in our cohort, we were not able to differentiate between antibody-mediated rejection (ABMR) and T-cell-mediated rejection (TCMR). Based on the individual variations in Th1- and Th17-associated cytokines, we propose an overlapping tissue response during ABMR and TCMR. Based on the weak Th1- and Th17-associated patterns in EMBs, indicative of cellular rejection, we focused primarily on AR. Yet, we found a potential contribution of circulating DSAs in recipient plasma to the micromilieu in myocardial tissue in later phases post HTx. In this regard, and as previously shown by us in kidney biopsies, CXCL9 and CXCL10 protein levels were also detected as potential biomarkers for ABMR (23). Based on these observations, elevated CXCL9 and CXCL10 concentrations are likely to represent a shared tissue damage and endothelial injury response plus immune cell infiltration across different organs during AR. However, further prospective studies are warranted to finally prove this hypothesis.

### 4.1 Limitations

The limitations of our study are a single-center cohort of heart transplant recipients, the variability in timing of sample collection posttransplant, and the lack of a larger cohort of EMBs, especially with higher rejection grades (2R, 3R). Moreover, the cohort of plasma samples with paired biopsies should be enlarged. Yet, this sample overlap between plasma and biopsies, as well as its paired analysis, introduces potential confounders and statistical dependence despite the lack of correlation between tissue and plasma soluble immune protein levels.

In terms of the experimental setup, EMB lysates were adjusted to total protein concentrations of 250 or 500 µg/ml (corresponding to 12.5/25 µg total protein per assay), whereas plasma proteins were not normalized, resulting in a relative comparison between these compartments.

### 4.2 Conclusion

In conclusion, we were able to identify a core chemokine signature for AR in EMB tissue composed of higher CXCL9, CXCL10, CCL3, and CCL4 concentrations. In contrast to this local environment within heart tissue, plasma cytokines and chemokines do not seem to be reliable circulating biomarkers, since we could not confirm this core signature in paired blood samples of HTx patients. These observations may help to identify clinical approaches for supporting histopathology and rejection treatment as well as improvements to the early diagnosis of AR in HTx recipients and individualized adjustment of immunosuppression.

### 4.3 Future directions

Based on our findings, we believe EMBs remain an important diagnostic procedure in the near future for the diagnosis of cardiac AR, since current diagnostics still seem to be insufficient to completely replace this tissue examination. However, several promising approaches have been suggested in the past including AI. To reduce interobserver variability in determining rejection states in EMBs, machine learning (ML) algorithms for automated interpretation of biopsy-based gene expression profiles (11) or whole-slide EMB images (14) have been developed. Moreover, several non-invasive alternatives, so-called liquid biopsies [i.e., dd-cfDNA (20), microRNA (45), piRNA (46)], have been implicated, with the relatively costly quantification of dd-cfDNA being already routinely applied in some hospitals. Lastly, non-invasive imaging techniques [i.e., CT-based assessment of pericoronary fat attenuation (47)] are promising approaches for early prediction of allograft rejection. Nevertheless, future research is also needed with the help of AI to translate these approaches into clinical routine.

## Data Availability

The raw data supporting the conclusions of this article will be made available by the authors, without undue reservation.
