# Supplementary material for: Core signature of rejection-associated cytokines and chemokines in endomyocardial biopsies after heart transplantation
Source: Front Cardiovasc Med. 2025 Aug 8;12:1612258. doi: 10.3389/fcvm.2025.1612258 (PMC12370642; doi:10.3389/fcvm.2025.1612258)
Supplement: Supplementary file 1 [file Datasheet1.pdf]

Supplementary Material

Core Signature of Rejection-Associated Cytokines and Chemokines in Endomyocardial Biopsies after Heart Transplantation

Lena M.-L. Radomsky, Jenny F. Kühne, Kerstin Beushausen, Jana Keil, Ludmilla Knigina, Yves Scheibner, Adelheid Görler, Arjang Ruhparwar, Fabio Ius, Christoph L. Bara, Christine S. Falk*

*** Correspondence:** Christine S. Falk: falk.christine@mh-hannover.de

Table of Content

	Page
Figure S1	3
Figure S2	4
Figure S3	5
Figure S4	6
Figure S5	7
Figure S6	8
Table S1	9
Table S2	10
Table S3	11

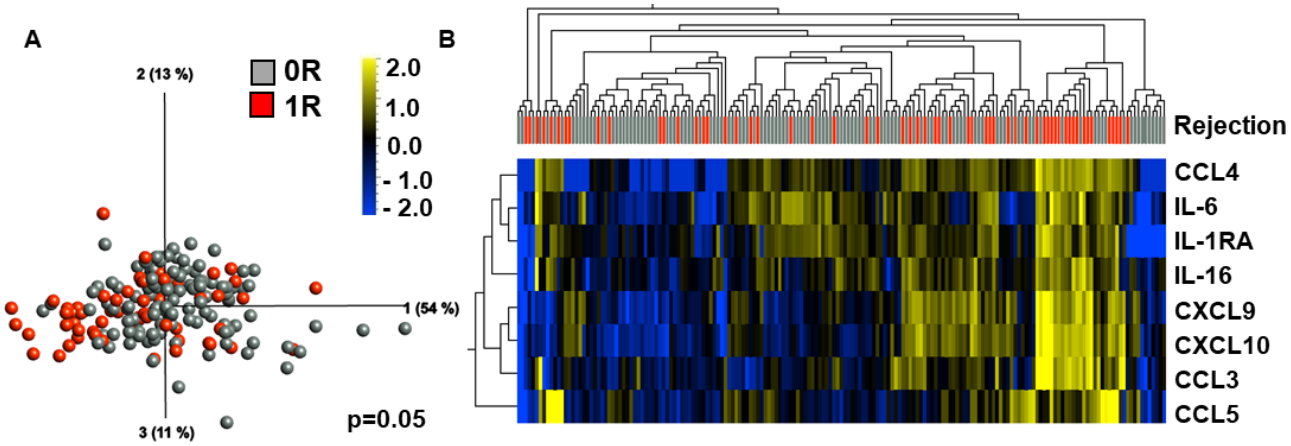

Figure S1: Concentrations of soluble mediators in heart biopsy lysates are significantly higher in 1R as compared to 0R tissue samples. EMBs were processed and cytokine/chemokine concentrations determined as described in Figure 1. (**A**) Principal component analysis of the 50 measured proteins according to 0R or 1R (0R, n= 121, grey; 1R/2R n=58, red) (*P*= 0.05 and q=0.279) and (**B**) unsupervised hierarchical clustering are shown. Two-group comparisons were used to identify variables differentially expressed between the two groups. Blue color indicates lower, yellow color indicates higher expression.

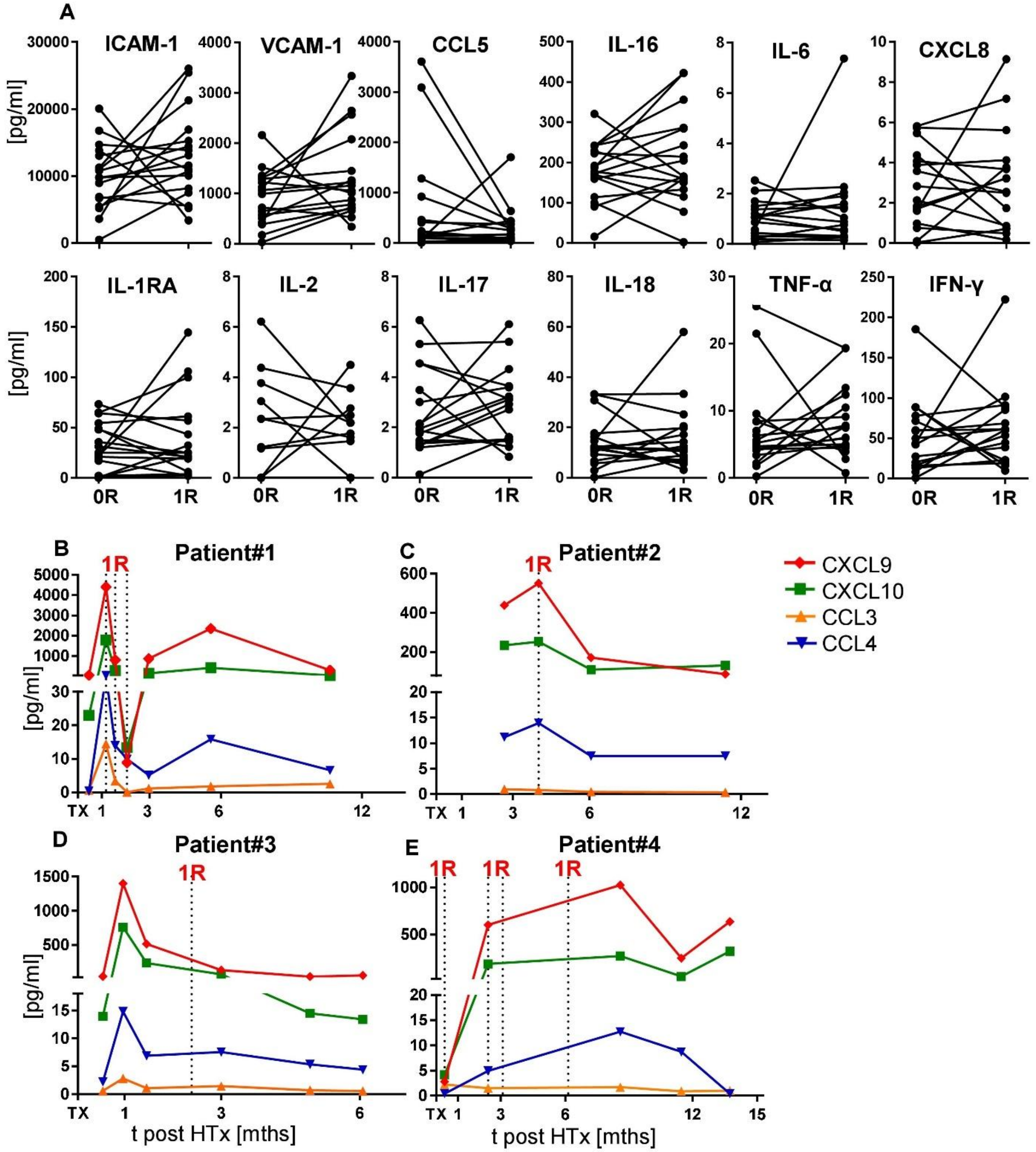

Figure S2: Individual patient development of concentrations over time after transplantation. Endomyocardial biopsies were procured and cytokine/chemokine concentrations determined as described in Figure 1. (**A**) Paired t-test (Wilcoxon–test) was applied on two consecutive biopsies with the second biopsy classified as histopathologic acute mild rejection (1R). Asterisks indicate *P* values with **P* < .05 ***P* < .01, ****P* < .001, *****P* < .0001 (n=17). (**B-E**) Individual protein concentrations in EMB lysates over time are displayed for four patients. Respective histopathological secured rejections are indicated (1R).

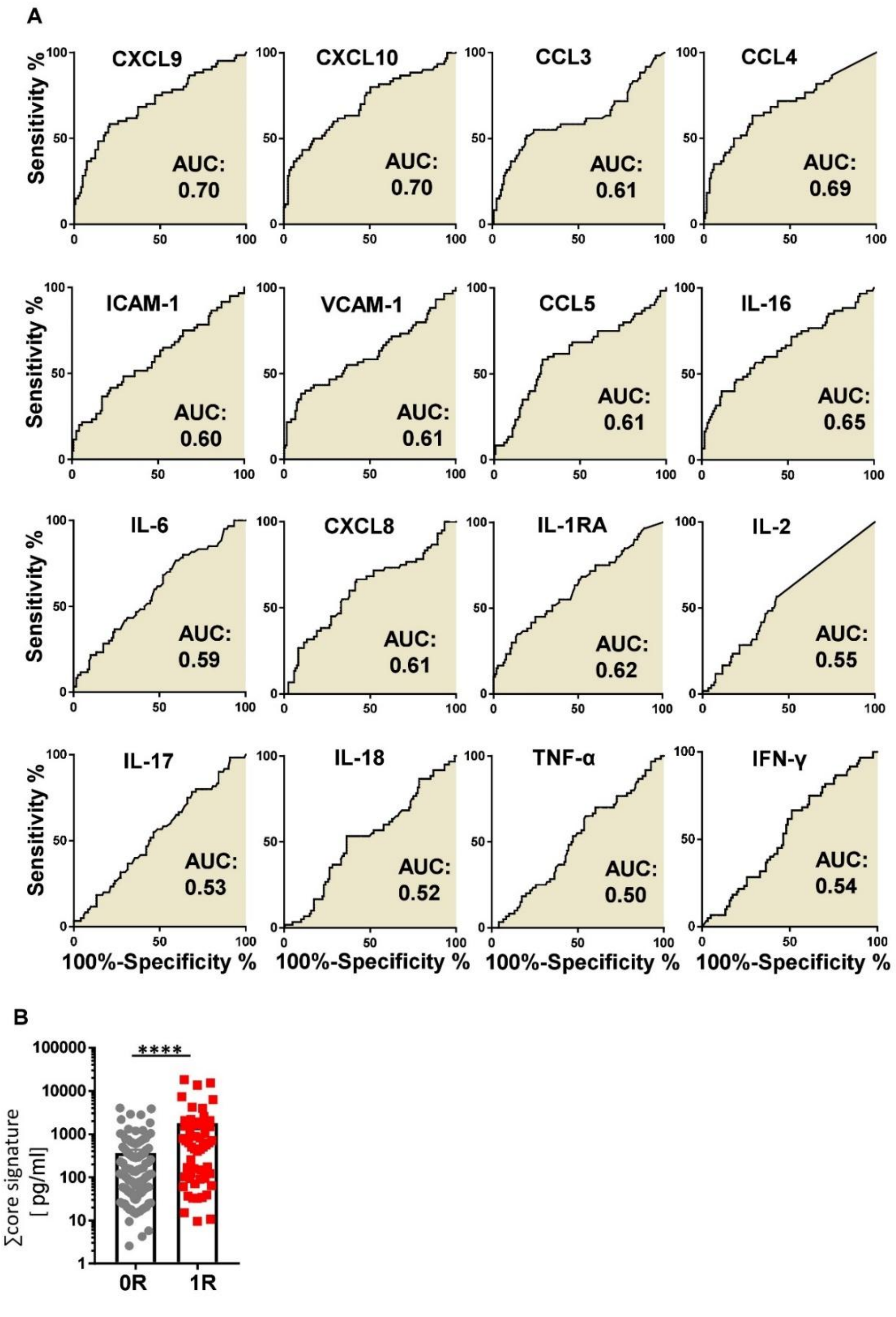

Figure S3: Combination of core signature chemokines is able to discriminate between rejection and no-rejection. EMBs were processed and cytokine/chemokine concentrations determined as described in Figure 1. (**A**) Receiver operating characteristics curve and area under the curve were calculated for the prediction of 0R vs. 1R/2R based on the quantified cytokine/chemokine concentrations in tissue lysates (0R, n= 121; 1R/2R, n=60). (**B**) Cumulative concentrations of the core signature chemokines CXCL9, CXCL10, CCL3 and CCL4 were calculated for samples without signs of acute rejection (0R, n=121, grey) and with histopathologic-confirmed acute rejection (1R, n=58, red). A two-tailed, unpaired *t*-test (Kruskal-Wallis) was applied for statistical analysis. Data are shown as mean ± SEM, asterisks indicate *P* values with ****$P < .0001$.

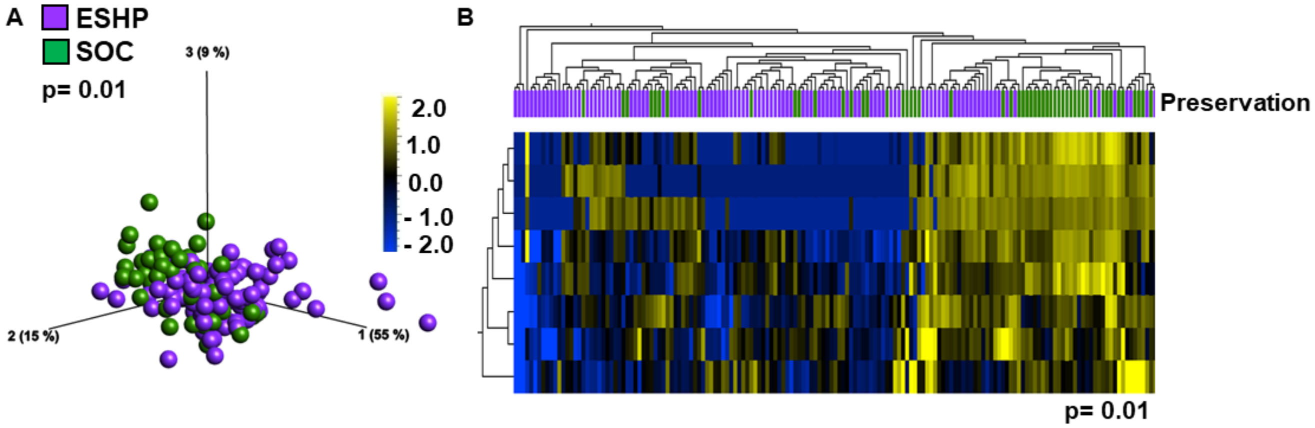

Figure S4: Slightly elevated cytokine/chemokine concentrations in tissue lysates of organs preserved with cold standard of care procedure as compared to *ex situ* heart perfusion. EMBs were processed and cytokine/chemokine concentrations determined as described in Figure 1. Here, only biopsies obtained during the first year after transplantation were included in the analyses. (**A**) Principal component analysis of the 50 measured proteins (P= 0.01 and q=0.05) and (**B**) unsupervised hierarchical clustering according to preservation method (ESHP, n=108, purple; SOC, n=53, green) are shown. Two-group comparisons were used to identify variables differentially expressed between the two groups. Blue color indicates lower, yellow color indicates higher expression.

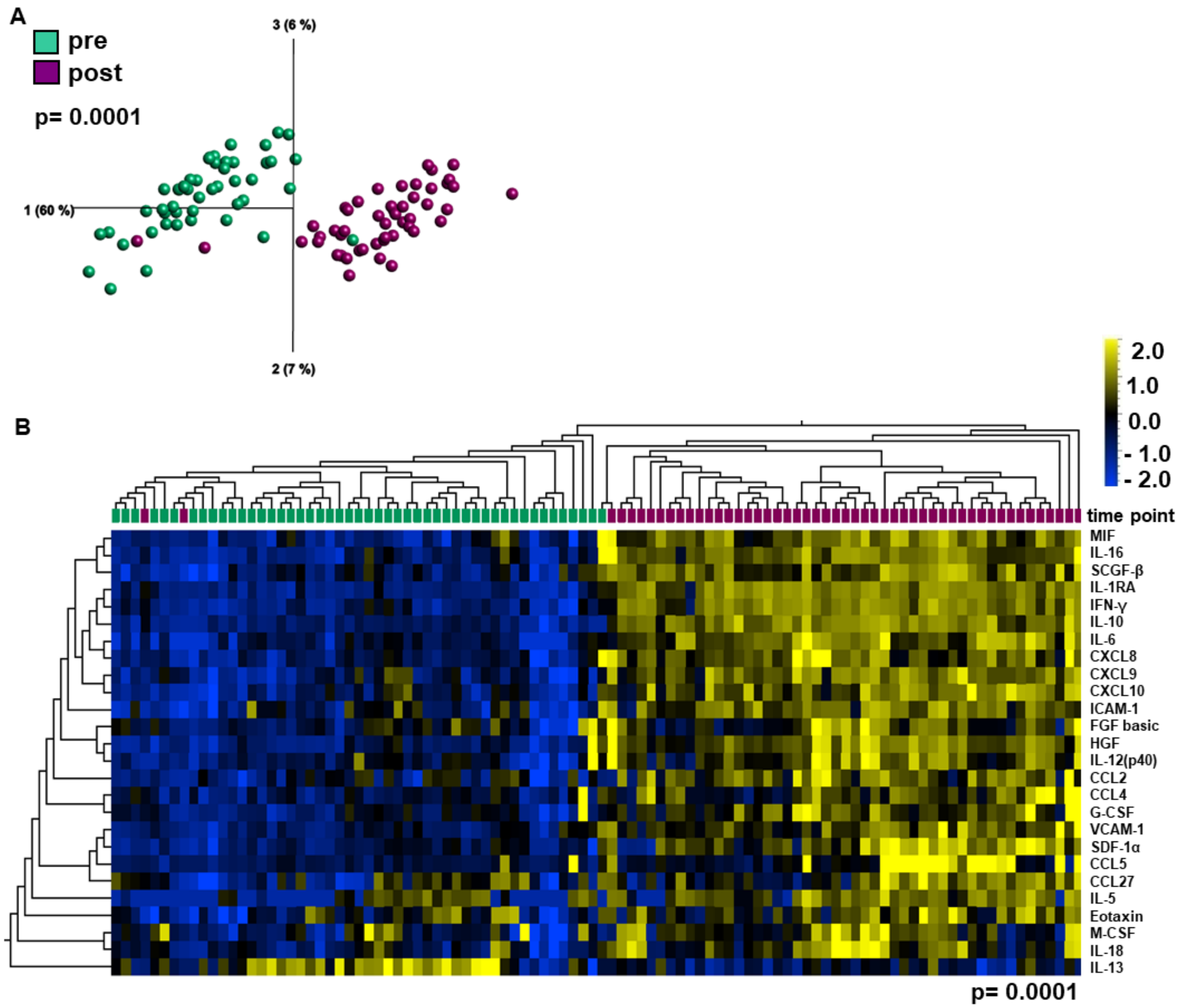

Figure S5: Ischemia reperfusion pattern in plasma samples consisting of elevated cytokine/chemokine concentrations. Plasma samples were isolated from peripheral blood obtained directly before (pre) or directly after (post) HTx followed by quantification of 50 different soluble mediators by Luminex-based multiplex assays. (**A**) Principal component analysis and (**B**) unsupervised hierarchical clustering of the 50 measured proteins according to sampling time point (pre, n=49, green; post, n=51, purple) (*P*= 0.0001 and q=1.44e^{-4}) are shown. Two-group comparisons were used to identify variables differentially expressed between the groups.

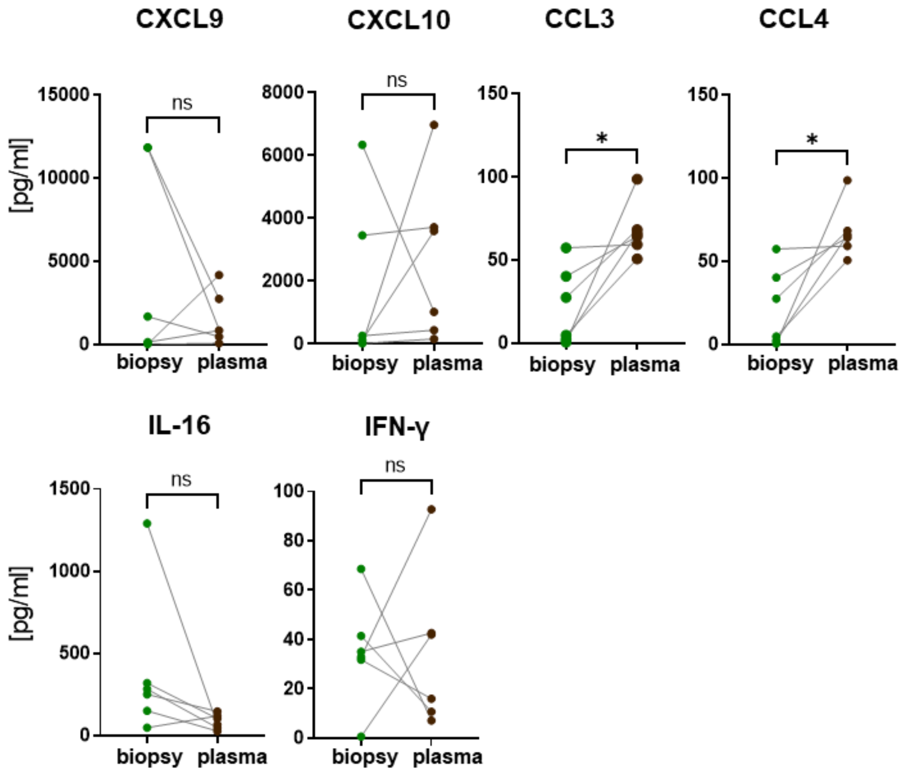

Figure S6: Distinct microenvironment in paired plasma samples and biopsy lysates (1R). Biopsies were taken and cytokine/chemokine concentrations determined as described in Figure 1. Here, only biopsies without histopathologic signs of acute rejection (1R) were included in the analysis and compared to the concentrations of the same 50 proteins quantified in paired peripheral blood plasma samples obtained ± 8d to EMB procurement (biopsy, n=6, green; plasma, n=6, brown). Data are shown as mean ± SEM. For statistical analysis, a two-tailed Wilcoxon matched-pairs signed rank test was applied. Asterisks indicate P values with *P < .05.

Table S1: Summary of normality testing (Shapiro-Wilk test) for core signature chemokines in 0R and 1R/2R biopsies, respectively. Table in support of findings displayed in Fig. 2 (0R, n=121, 1R/2R, n=60).

	**0R**			**1R + 2R**		
	P value*	Passed normality test	P value summary	P value*	Passed normality test	P value summary
**CXCL9**	<0.0001	no	****	<0.0001	no	****
**CXCL10**	<0.0001	no	****	<0.0001	no	****
**CCL3**	<0.0001	no	****	<0.0001	no	****
**CCL4**	<0.0001	no	****	<0.0001	no	****

Table S2: No influence of bridge to transplant prior to HTx on cytokine/chemokine concentrations. EMBs were processed and cytokine/chemokine concentrations determined as described in Figure 1. Here, only the first biopsy per patient obtained during the first 26 days after transplantation was considered for the analysis. On the basis of clinical patient data two groups were formed (no-BTT, n=8; BTT, n=27) and unpaired t-test (Mann-Whitney) was applied. Data are mean values ± standard deviation.

	**no-BTT** n=8 [pg/ml]	**BTT** n=27 [pg/ml]	**p-value**
**CXCL9**	994 ± 2101	1074 ± 3145	0,45
**CXCL10**	267,5 ± 441	462.7 ± 1357	0,22
**CCL3**	2,4 ± 2,5	3,9 ± 6,6	0,75
**CCL4**	13,1 ± 11,9	12,3 ± 14,5	0,62
**ICAM-1**	11047 ± 9600	11848 ± 5947	0,25
**V-CAM-1**	1229 ± 1167	1058 ± 667,9	0,89
**CCL5**	238,5 ± 254,6	1085 ± 2882	0,36
**IL-16**	261,4 ± 133,1	198,6 ± 230,7	0,051
**IL-6**	1,3 ± 0,9	1,3 ± 1,5	0,38
**CXCL8**	3,0 ± 2.0	8,8 ± 19,4	0,63
**IL-1RA**	34,0 ±39,6	52,5 ±61,8	0,34
**IL-2**	1,8 ± 1,8	1,1 ± 2,2	0,16
**IL-17**	3,1 ± 2,2	2,4 ± 1,9	0,37
**IL-18**	12,6 ± 7,1	12,4 ± 6,2	0,86
**TNF-α**	5,3 ± 3,6	6,8 ± 4,8	0,45
IFN-γ	30,8 ± 15,3	44,4 ± 31,9	0,45

Table S3: No difference in paired plasma concentrations comparing 0R and 1R samples. Cytokine/chemokine concentrations for plasma samples obtained at different time-points after HTx that match EMB procurement (± 8d) are shown based on histopathologic classification of the respective biopsy as no signs of acute rejection (0R, n=37) vs. acute mild rejection (1R, n=10) are shown. For statistical analyses unpaired t-test (Mann-Whitney test) was used. Data are mean values ± standard deviation (SD). BTT: Bridge to transplant

	0R<br>n=37 [pg/ml]	1R<br>n=10 [pg/ml]	p-value
CXCL9	266,5 ± 684	542 ± 835,3	0,18
CXCL10	409,4 ± 1131	963,2 ± 1448	0,17
CCL3	13,37 ± 59,88	6,75 ± 7,05	0,14
CCL4	64,22 ± 45,09	58,12 ±1 0,68	0,58
ICAM-1	34141 ± 207006	37822 ± 20610	0,58
V-CAM-1	25240 ± 11599	29722 ± 19431	0,89
CCL5	3299 ± 1757	3100 ± 1238	0,97
IL-16	90,51 ± 65,38	80,5 ± 40,42	0,87
IL-6	36,16 ± 206,3	7,44 ± 11,23	0,14
CXCL8	5,847 ± 5,7	8,7 ± 8,39	0,43
IL-1RA	117,9 ± 154,1	291,5 ± 444,5	0,098
IL-2	2,62 ± 3,77	2,78 ± 3,56	0,96
IL-17	5,13 v 3,12	5,56 ± 2,76	0,65
IL-18	56,66 ± 89,58	85,3 ± 135,9	0,29
TNF-α	16,7 ± 9,51	18,76 ± 9,44	0,42
IFN-γ	14,17 ± 10,14	21,98 ± 27,04	0,7
IL-4	1,143 ± 0,9945	1,284 ± 0,9306	0,48
IL-1β	3,387 ± 4,331	3,699 ± 2,614	0,24
IL-5	10,79 ± 14,48	13,12 ± 15,9	0,88
IL-7	8,742 ± 4,628	10,01 ± 5,249	0,5
IL-9	65,31 ± 23,12	66,37 ± 14,75	0,57
IL-10	6,185 ± 4,232	7,273 ± 5,377	0,71
IL-12 (p70)	2,111 ± 2,04	2,196 ± 1,89	0,82
IL-13	6,984 ± 7,601	7,825 ± 5,588	0,36
IL-15	34,58 ± 33	39,7 ± 29,86	0,57
CCL11	24,77 ± 13,79	30,67 ± 28,38	0,99
FGF basic	11,82 ± 6,117	13,17 ± 7,541	0,76
G-CSF	97,11 ± 187,8	113 ± 107	0,34
GM-CSF	2,432 ± 2,817	3,256 ± 3,353	0,32
CCL2	15,97 ± 13,74	38,11 ± 34,09	0,01
PDGF-bb	216 ± 86,84	222,3 ± 86,67	0,75
VEGF	42,2 ± 36,83	50,95 ± 30,75	0,32
CCL27	451,2 ± 277,1	472,6 ± 256,7	0,61
CXCL1	563,9 ± 197,9	651,5 ± 183	0,12
HGF	338,3 ± 737,6	507,5 ± 573,1	0,31
IFN-α2	3,856 ± 2,367	4,529 ± 2,397	0,4
IL-3	0,57 ± 0	0,57 ± 0	>0,99
IL-1α	23,07 ± 14,21	28,76 ± 18,49	0,4
IL-12 IL-23 (p40)	301,3 ± 226,5	343,4 ± 212,1	0,55
IL-18	56,66 ± 89,58	85,3 ± 135,9	0,29
LIF	40,25 ± 30,91	45,55 ± 30,47	0,53
CCL7	2,638 ± 2,842	35,8 ± 105,2	0,68
M-CSF	48,17 ± 36,35	64,31 ± 54,13	0,47
MIF	2446 ± 1071	2076 ± 1002	0,39
ß-NGF	0,5403 ± 0,4001	0,584 ± 0,3697	0,60
SCF	83,28 ± 65,41	98,56 ± 88,78	0,93
SCGF-ß	236867 ± 111860	264635 ± 140915	0,64
CXCL12	140,7 ± 54,7	120,6 ± 36,29	0,31
TRAIL	41,71 ± 13,96	62,05 ± 73,85	0,71
IL-2Rα	83,98 ± 71,35	143,1 ± 252,5	0,94
